# Effects of low-flow sevoflurane anesthesia on renal function in low birth weight infants

**DOI:** 10.1186/1471-2253-15-6

**Published:** 2015-01-21

**Authors:** Na Xing, Xin Wei, Yanzi Chang, Yingying Du, Wei Zhang

**Affiliations:** Department of Anesthesiology, The First Affiliated Hospital of Zhengzhou University, Zhengzhou, 450052 China

**Keywords:** Low-flow, Inhalation anesthetics, Low birth weight infants, Renal function

## Abstract

**Background:**

Low-flow sevoflurane anesthesia has been shown to influence renal function in rats, but not in adult humans. Presently, no study has assessed the effects of sevoflurane on renal function in low birth weight infants. Our aim was to study the renal function in low birth weight infants undergoing surgery with low-flow sevoflurane anesthesia.

**Methods:**

Forty infants graded as American Society of Anesthesiologists (ASA) grade I or II undergoing abdominal surgery were selected. After the induction of anesthesia, they received sevoflurane semi-closed inhalation anesthesia with an oxygen flow rate of 1 L/minute. According to patient vital signs, in-tidal sevoflurane concentration was maintained at 2.5%–4.0%. Peripheral vein blood samples and urine specimens were obtained before surgery (T_0_), at the end of surgery (T_1_), and 24 (T_2_), 48 (T_3_), and 72 hours (T_4_) after surgery. Serum creatinine (Cr), blood urea nitrogen (BUN), urinary retinol binding protein (RBP), and β-N-acetyl-glucosaminidase (NAG) levels were determined at these time points. Also, a temperature probe was inserted into the center of a soda lime canister and temperature readings were obtained.

**Results:**

There were no significant differences in Cr and BUN before and after surgery (*P* > 0.05). However, RBP and NAG levels increased after surgery (*P* < 0.05), but returned to preoperative levels 72 hours (T_4_) after surgery. The highest soda lime temperature was 37.3 ± 3.1°C.

**Conclusions:**

Low-flow sevoflurane semi-closed inhalation anesthesia has no significant effect on the renal function of low birth weight infants.

## Background

Due to its low blood gas partition coefficient (0.69) and neutral odor, sevoflurane (S) is suitable for inhalational anesthesia in pediatric patients [1]. Low-flow anesthesia has been widely used in adult anesthetic practice as it significantly reduces the wastage of expensive volatile anesthetic agents [2]. S is degraded by carbon dioxide absorbents during low-flow anesthesia and forms the fluoride and haloalkene Compound A. Larger doses of compound A causes nephrotoxicity in rats that is characterized by corticomedullary proximal tubular necrosis, and evidenced by proteinuria, glucosuria, and enzymuria as well as increased serum creatinine (Cr) and blood urea nitrogen (BUN) [3, 4]. In adult patients, most related studies have found no clinically significant effect of low-flow sevoflurane on renal function, as evaluated by standard clinical measures (Cr and BUN) [5]. However, experience with S in low birth weight (LBW) infants is limited. The survival rate of LBW infants has increased with improvements in perinatal care, which has enabled more opportunities to undergo surgical procedures, and anesthesiologists must provide sensible anesthesia to these infants [6]. The purpose of this investigation, therefore, was to use conventional serum and urinary markers to assess the effects of low-flow (1 L/min) S anesthesia on renal function and clinical safety in LBW infants undergoing elective abdominal surgery.

## Methods

The study was registered with Chinese Clinical Trial Registry (registration no:ChiCTR-ONRC-14004771). And the investigational protocol was approved by the Ethics Committee of the Affiliated Hospital of Zhengzhou University (2013015). All parents provided their written informed consent. Forty ASA I or II infants (male n = 22, female n = 18, birth weight 1550–2495 g, gestational age 32–40 weeks, age 6–55 days) undergoing elective abdominal surgery with an expected operative time of 60–120 min were enrolled in this study. None of these patients had a history of respiratory distress syndrome, hepatic or renal disease, or abnormal kidney presentation under B-ultrasound.

### Examination

Infants who received nephrotoxic drugs (eg: antibiotics and antiviral drugs) before anesthesia were excluded. Included infant patients had preoperative breast milk restriction for 4 hours and fluid limitation for 2 hours prior to undergoing surgery. The patients were premedicated with 0.5 mg/kg of dexamethasone and 0.1 mg/kg of atropine intramuscularly 30 minutes before anesthesia induction. After entering the operation room, a venous access was established and an intravenous drip of 5% glucose injection was maintained at 10–20 mL/kg/hour. The temperature of the operating room was regulated at 26–30°C. The infants were monitored with noninvasive blood pressure, electrocardiogram, pulse-oximetry, end-tidal carbon dioxide, cutaneous temperature, and urine volume (weight of diapers).

The anesthetic protocol was similar to that used previously [7]. Anesthesia was induced by S inhalation at a concentration of 6%–8% (product batch number: 1312531; Jiangsu Hengrui Medicine Co Ltd, Lianyungang, People’s Republic of China) in 40%–80% oxygen at 5 L/min. When the infants lost the eyelash reflex, propofol 1 mg/kg, remifentanil 1 μg/kg, and cis-atracurium 0.1 mg/kg were intravenously injected. Next, tracheal intubation was performed followed by mechanical ventilation. After induction, the fresh gas flow rate was set at 1 L/minute with low-flow semi-closed S. Next, in-tidal S concentration was maintained at 2.5%–4.0% according to vital signs. Ventilation was controlled with airway pressure of 18–25 cm H_2_O. The breathing rate was set at 30–45 respirations/minute. PETCO2 levels were maintained at 30–40 mmHg. SpO_2_ was maintained at 85%–100%. Respirator parameters were adjusted according to blood gas analyses. Systolic arterial pressure (SAP) was maintained at 40–65 mmHg (1 mmHg = 0.133 kPa) and heart rate was maintained at 160–180 bpm. Approximately 10 minutes before the end of the surgery, S inhalation was closed. When the infants engaged in spontaneous breathing, the endotracheal tube was removed.

Peripheral vein blood samples and urine specimens were taken before surgery (T0), at the end of surgery (T1), and 24 (T2), 48 (T3), and 72 (T4) hours after surgery. Serum creatinine (Cr), blood urea nitrogen (BUN), urinary retinol binding protein (RBP), and β-N-acetyl-glucosaminidase (NAG) levels were tested. In addition, a temperature probe was inserted into the center of soda lime canister and the highest temperature readings were recorded. The colorimetry method was used to test serum Cr and BUN levels. The immunoturbidimetric method was used to test urinary RBP and NAG and each urine specimen was simultaneously tested for Cr. To avoid errors caused by urine concentration differences or dilutions, results are reported as the ratio of enzyme activity and urinary creatinine.

### Statistical analysis

Data were analyzed using SPSS 13.0 (SPSS Inc, Chicago, IL, USA). Results are expressed as means ± standard deviation (SD). Intragroup comparisons were performed using repeated measures analysis of variance (ANOVA). For all tests, a *P*-value <0.05 was considered statistically significant.

### Ethics statement

Written informed consent was obtained from the parents for publication of this article and any accompanying images, and obtained the permission of the Ethics Committee of the Affiliated Hospital of Zhengzhou University.

## Results

The characteristics of infants and the surgical procedures they underwent are listed in Table 1. The renal function (Cr and BUN) values increased at T1, T2, T3, and T4 compared with T_0_, but these increases were not significant, for Cr *P* = 0.062, 0.079, 0.116, 0.201, for BUN *P* = 0.104, 0.093, 0.112, 0.268. RBP concentrations increased at T1 compared with T_0_ (*P* = 0.041). At T2, T3, and T4, RBP levels decreased gradually, but not significantly (*P* = 0.110, 0.197, 0.234). NAG concentrations increased to a maximum value at T2 (*P* = 0.016), but returned to preoperative levels at T_4_ (*P* =0.112, 0.099, 0.206) (Table 2). The highest soda lime temperature was 37.3 ± 3.1°C.

**Table 1:** **Infant patient characteristics**

Characteristics	Value (range)
Gestational age (weeks) et al.	34.01 ± 2.12 (32–40)
Sex (male/female)	22/18
Birth weight (kg)	2.07 ± 0.35 (1.55-2.45)
Age (days)	26.5 ± 15.72 (6–55)
Weight (kg)	2.45 ± 0.69 (1.89-3.53)
Anesthesia time (min)	78.41 ± 12.98 (55–92)
Urine volume (ml/day)	85.7 ± 12.6(70–100)
Surgical procedure	
Congenital megacolon	7
Congenital intestinal atresia	5
Omphalitis	6
Congenital hypertrophic pyloric stenosis	6
Umbilical hernia	5
Anal atresia	6
Intussusception	5

**Table 2:** **Renal biomarker values**

Renal biomarkers	T _0_ (before)	T _1_ (end)	T _2_ (24 h)	T _3_ (48 h)	T _4_ (72 h)
Cr (μmol/L)	68.14 ± 15.45	73.78 ± 11.32	71.38 ± 12.43	69.50 ± 10.51	70.29 ± 13.41
*P* value	-	0.062	0.079	0.116	0.201
BUN (mmol/L)	3.82 ± 0.63	4.21 ± 0.71	4.56 ± 1.15	4.07 ± 1.03	3.98 ± 1.11
*P* value	-	0.104	0.093	0.112	0.268
RBP (mg/L)	0.24 ± 0.01	0.50 ± 0.02*	0.32 ± 0.08	0.31 ± 0.04	0.29 ± 0.03
*P* value	-	0.041	0.110	0.197	0.234
NAG (U/mmol · Cr)	10.85 ± 0.11	11.34 ± 3.42	15.21 ± 1.07*	12.69 ± 1.57	11.19 ± 1.12
*P* value	-	0.112	0.016	0.099	0.206

Values are means ± standard deviation; *significantly higher than before surgery (T_0_).

Cr = Creatinine; RBP = urinary retinol binding protein; BUN = blood urea nitrogen; NAG = N-acetyl-β-D-glucosaminidase.

## Discussion

Low birth weight infants are defined as infants born with a birth weight of less than 2,500 g, regardless of their term status [8], and in these infants, the kidneys are underdeveloped. However, the gestational age of infants in our study was 32–40 weeks, at which time renal function will be similar to normal birth weight infants, which justifies our decision to combine preterm and term infants in our analysis. To exclude the impact of the type of surgery on the results, patients were limited to those undergoing abdominal surgery. As low birth weight infants often have underdeveloped thermoregulatory centers, they are susceptible to hypothermia. Therefore, we regulated the temperature of the operating room at 26–30¡ãC and heated intraoperative blood transfusion fluid and rinse solutions to maintain infant body temperatures above 36¡ãC. Low birth weight infants have lower blood volume, and even blood loss of as little as 10–20 mL can cause hypovolemic shock [8]. According to the pediatric anesthetic protocols of our unit, during the first hour of surgery, 5% glucose was infused at a rate of 10–20 mL/kg. The indications for transfusion were blood losses of >10% blood volume or a hematocrit <30%. No patients in our population suffered massive hemorrhage or hypovolemia shock during surgery, and no blood transfusions or perioperative diuretics were required. Furthermore, intramuscular atropine before anesthesia was used to dry the airways and reduce excitability of the vagus nerve to prevent bradycardia. Intramuscular dexamethasone, on the other hand, was used to effectively prevent laryngeal edema and allergic reactions. In our study, the pressure control ventilation mode was selected during surgery to avoid lung injury caused by high pressure ventilation. Moreover, prolonged high-flow oxygen inhalation is one of the most dangerous factors for retinopathy [9]; therefore, we set the semi-closed S flow to 1 L/minute in 40%–80% oxygen. In our study, none of the infants become hypoxic and all had consistently stable vital signs during surgery, which were highly important for the renal response to low-flow anesthesia.

S is a nonpungent inhalation general anesthetic agent that is degraded by soda lime to sequester carbon dioxide in the anesthesia breathing circuit. The main degradation product of S is pentafluoroisopropenyl fluoromethyl (PIFE/Compound A), which is nephrotoxic in rats as a result of beta -lyase-mediated metabolism [3]; however, S is not associated with nephrotoxicity in adults. The safety of low-flow S anesthesia with respect to renal function of low birth weight infants is determined by the effect of Compound A. Based on previous findings [10], and results from our preliminary experiment, we set the fresh gas flow rate of semi-closed S anesthesia to 1 L/minute.

Serum Cr and BUN are standard tests for renal function. Based on previous literature [11], we used absolute values of serum Cr and BUN at predefined time points (T1-T4) as biomarkers to assess renal injury due to the potential nephrotoxicity of Compound A. Our results demonstrate that there were no significant differences in Cr and BUN before and after surgery, which is consistent with a study by Fukud et al. [11]. What’ s more, Cr levels in the first two weeks after birth are strongly dependent on the maternal creatinine levels. The reason why low-flow S anesthesia produces minimal Compound A concentrations might be related to the fact that there is low β-lyase activity in low birth weight infants relative to rats [12]. While composition, temperature, humidity of CO_2_ absorbent, and concentration of inhalation anesthetic agent may affect the concentration of Compound A exposure during ventilation, fresh gas flow is the most important factor [13]. Osawa et al. considered that Compound A concentration in blood and soda lime temperature was positively correlated [14]. In our study, a temperature probe was inserted into the center of a soda lime canister. The highest temperature of the soda lime remained 37.3 ± 3.1°C during surgery, which is lower than the 44.9°C measured by Yu Rongguo et al., and indicative of low Compound A concentrations [15].

RBP and NAG were measured in our study because they were sensitive biomarkers of nephrotoxicity and have been widely used in several S clinical studies [16]. RBP are blood proteins that are used to transport retinoids that are filtered in the glomeruli. Almost all RBP (99.7%) are reabsorbed by the proximal convoluted tubule, while only a very small amount is excreted in the urine. RBP has better stability in an acidic environment than beta 2 microglobulin (β2MG) and thus can reflect early renal tubule damage [17]. NAG, on the other hand, is mainly excreted by renal proximal tubule epithelial cells that is rarely detectable in urine under normal conditions. When renal tubules are dysfunctional, such as when tubular epithelial cells rupture, NAG is released into the urine and thus NAG activity can be used as a possible biomarker of kidney damage [18]. Our study demonstrated increased levels of RBP and NAG postoperatively (T1) and 24 (T2) hours after surgery, but these levels returned to preoperative baselines 72 (T4) hours after surgery, which is consistent with the results of the study by Ryu et al. [2]. The elevated levels of these proteins may be a result of surgical stress or antibiotics, which leads to a massive release of enzymes into the urine and tubular reabsorption dysfunction and, as a result, increased RBP and NAG levels. Another reason for these increases may be due to Compound A or other metabolic products combining with tubular proteins resulting in temporary renal dysfunction, which is consistent with the results reported by Kobayashi et al. [19].

### Limitations

First, because there was no control group (isoflurane or desflurane), the results could be influenced also by the surgery itself. Second, it has been reported that during the first 2 hours of low-flow S anesthesia, Compound A concentrations could increase before falling 4 hours postoperatively [4]. In our study, the mean anesthetic time was 78 minutes. Therefore, we could not ascertain the impact of prolonged S inhalation on the renal function of LBW infants in this study. Moreover, although plasma creatinine and BUN levels are sensitive markers for renal dysfunction, other markers are more sensitive. These include cystatin c and NGAL levels.

## Conclusions

The results suggest that low- flow S anesthesia did not significantly influence renal function in low birth weight infants undergoing abdominal surgery.

## Abbreviations

ANOVA: Analysis of variance
SD: Standard deviation
PIFE/Compound A: Pentafluoroisopropenyl fluoromethyl
Cr: Serum creatinine
BUN: Blood urea nitrogen
RBP: Urinary retinol binding protein
NAG: β-N-acetyl-glucosaminidase
S: Sevoflurane.

## References

[1] Oofuvong M, Siripruekpong S, Naklongdee J, Hnookong R, Lakateb C (2013). Comparison the incidence of emergence agitation between sevoflurane and desflurane after pediatric ambulatory urologic surgery. J Med Assoc Thai.

[2] Ryu HG, Lee JH, Lee KK, Gil NS, Kim CS, Sim SE (2011). The effect of low fresh gas flow rate on sevoflurane consumption. Korean J Anesthesiol.

[3] Kharasch ED, Schroeder JL, Sheffels P, Liggitt HD (2005). Influence of sevoflurane on the metabolism and renal effects of compound A in rats. Anesthesiology.

[4] Kharasch ED, Frink EJ, Artru A, Michalowski P, Rooke GA, Nogami W (2001). Long-duration low-flow sevoflurane and isoflurane effects on postoperative renal and hepatic function. Anesth Analg.

[5] Wujtewicz M, Sawicka W, Wenski W, Marciniak A, Wujtewicz MA, Stepnowski P (2012). The influence of low flow anaesthesia on renal function in cancer patients previously treated with nephrotoxic chemotherapeutic agents. Anaesthesiol Intensive Ther.

[6] Keiko K (2004). Anaesthetic considerations for the management of very low and extremely low birth weight infants. Best Prac Res Clin Anaesthesiol.

[7] Chandler JR, Myers D, Mehta D, Whyte E, Groberman MK, Montgomery CJ (2013). Emergence delirium in children: a randomized trial to compare total intravenous anesthesia with propofol and remifentanil to inhalational sevoflurane anesthesia. Paediatr Anaesth.

[8] White SL, Perkovic V, Cass A, Chang CL, Poulter NR, Spector T (2009). Is low birth weight an antecedent of CKD in later life? A systematic review of observational studies. Am J Kidney Dis.

[9] Soares CR, Silveira RC, Procianoy RS (2010). Ophthalmic artery blood flow in very-low-birth-weight preterm infants. Invest Ophthalmol Vis Sci.

[10] Obata R, Bito H, Ohmura M, Moriwaki G, Ikeuchi Y, Katoh T (2000). The effects of prolonged low-flow sevoflurane anesthesia on renal and hepatic function. Anesth Analg.

[11] Kanbak M, Karagoz AH, Erdem N, Oc B, Saricaoglu F, Ertas N (2007). Renal safety and extrahepatic defluorination of sevoflurane in hepatic transplantations. Transplant Proc.

[12] Katayama R, Nagata S, Iida H, Yamagishi N, Yamashita T, Furuhama K (2011). Possible role of cysteine-S-conjugate β-lyase in species differences in cisplatin nephrotoxicity. Food Chem Toxicol.

[13] Marini F, Bellugi I, Gambi D, Pacenti M, Dugheri S, Focardi L (2007). Compound A, formaldehyde and methanol concentrations during low-flow sevoflurane anaesthesia: comparison of three carbon dioxide absorbers. Acta Anaesthesiol Scand.

[14] Osawa M, Shinomura T (1998). Compound A concentration is decreased by cooling anesthetic circuit during low flow Sevoflurane anesthesia. Can J Anesth.

[15] Rongguo Y, Li C, Lin S, Wang Y, Yang L, Lin Y (2000). Effects of low-flow sevoflurane anesthesia on renal tubular function. J Clin Anesthesiol.

[16] Hong JD, Lim IS (2012). Correlation between glomerular filtration rate and urinary N acetyl-beta-D glucosaminidase in children with persistent proteinuria in chronic glomerular disease. Korean J Pediatr.

[17] Hu C, Yang DP, Xu K, Cao H, Wu B, Cui D (2012). Ag@BSA core/shell microspheres as an electrochemical interface for sensitive detection of urinary retinal-binding protein. Anal Chem.

[18] Wu ZJ, Huang SM, Chen R, Hu B, Chen Y, Zhu YP (2009). Value of blood apoH gene expression and urinary NAG and RBP in early diagnosis of renal function damage in neonates. Zhongguo Dang Dai Er Ke Za Zhi.

[19] Kobayashi S, Bito H, Obata Y, Katoh T, Sato S (2003). Compound A concentration in the circle absorber system during low-flow sevoflurane anesthesia: comparison of Drägersorb Free, Amsorb, and Sodasorb II. J Clin Anesth.

[CR20] The pre-publication history for this paper can be accessed here: http://www.biomedcentral.com/1471-2253/15/6/prepub

